# Impact of metadata in multimodal classification of bone tumours

**DOI:** 10.1186/s12891-024-07934-9

**Published:** 2024-10-19

**Authors:** Florian Hinterwimmer, Michael Guenther, Sarah Consalvo, Jan Neumann, Alexandra Gersing, Klaus Woertler, Rüdiger von Eisenhart-Rothe, Rainer Burgkart, Daniel Rueckert

**Affiliations:** 1 Department of Orthopaedics and Sports Orthopaedics, Klinikum rechts der Isar, Technical University of Munich, Trogerstraße 26, 81675 Munich, Germany; 2 Institute for AI and Informatics in Medicine, Technical University of Munich, Munich, Germany; 3 Musculoskeletal Radiology Section, Klinikum rechts der Isar, Technical University of Munich, Munich, Germany; 4 Department of Computing, Imperial College London, London, UK

**Keywords:** Radiography, Bone neoplasm, Classification, Deep learning, Metadata

## Abstract

**Abstract:**

The accurate classification of bone tumours is crucial for guiding clinical decisions regarding treatment and follow-up. However, differentiating between various tumour types is challenging due to the rarity of certain entities, high intra-class variability, and limited training data in clinical practice. This study proposes a multimodal deep learning model that integrates clinical metadata and X-ray imaging to improve the classification of primary bone tumours. The dataset comprises 1,785 radiographs from 804 patients collected between 2000 and 2020, including metadata such as age, affected bone site, tumour position, and gender. Ten tumour types were selected, with histopathology or tumour board decisions serving as the reference standard.

**Methods:**

Our model is based on the NesT image classification model and a multilayer perceptron with a joint fusion architecture. Descriptive statistics included incidence and percentage ratios for discrete parameters, and mean, standard deviation, median, and interquartile range for continuous parameters.

**Results:**

The mean age of the patients was 33.62 ± 18.60 years, with 54.73% being male. Our multimodal deep learning model achieved 69.7% accuracy in classifying primary bone tumours, outperforming the Vision Transformer model by five percentage points. SHAP values indicated that age had the most substantial influence among the considered metadata.

**Conclusion:**

The joint fusion approach developed in this study, integrating clinical metadata and imaging data, outperformed state-of-the-art models in classifying primary bone tumours. The use of SHAP values provided insights into the impact of different metadata on the model’s performance, highlighting the significant role of age. This approach has potential implications for improving diagnostic accuracy and understanding the influence of clinical factors in tumour classification.

## Background

Bone tumours encompass various tumour entities, with the vast majority being benign [1, 2]. Malignant primary bone tumours, though accounting for a mere 0.2% of adult malignancies [3], rank as the sixth most common cancer in children and the third most common in adolescents [4]. Diagnosis in the early stages is challenging due to the absence of specific symptoms, resulting in significant treatment delays [5]. Timely referral to specialized tumour centres is crucial for comprehensive evaluation and differentiation between benign, intermediate, and malignant tumours [2]. Unfortunately, non-oncology-trained medical professionals encounter only a few malignant primary bone tumours throughout their careers, leading to potential delays of over a year and a lack of experience in identifying these complex tumour entities unequivocally [6]. In 2018, the Musculoskeletal Tumour Society and American Academy of Orthopedic Surgeons Working Group recommended plain radiographs as the initial screening for bone tumours [7], including for children [8]. Even if only a radiograph is available, patients with suspected malignant lesions should be referred to musculoskeletal tumour centres to prevent treatment delays. The need for further imaging studies should be assessed at referral centres [7]. The final diagnosis relies on synthesizing clinical presentation, imaging features, and histopathologic findings if a specific radiologic diagnosis of a benign entity proves inconclusive [9].

The field of diagnostic imaging is rapidly advancing, with technology, innovation, and market expansion leading to increased production of imaging and clinical data [10]. Precision medicine plays an increasingly important role in musculoskeletal radiology and orthopaedic oncology, necessitating advanced analysis tools [1, 11–14]. While artificial intelligence (AI), specifically Deep Learning (DL), is widely employed in lung, breast, and CNS cancer research [15], its application in musculoskeletal tumour research remains limited [1]. Nevertheless, these advanced data analysis techniques hold the potential to revolutionize the medical field, benefiting both physicians and patients [16]. In this study, we propose a DL model that emulates expert radiologists by incorporating clinical metadata alongside imaging data for diagnostic assessment and dataset enrichment. By doing so, our research question aligns closer with clinical reality: Can a state-of-the-art DL model accurately classify ten different bone tumour entities, leveraging the inclusion of clinical metadata from patients and providing insights into the significance of each clinical parameter?

## Methods

This retrospective study (N°48/20S) was approved by the local institutional review and ethics board, following national and international guidelines. Informed consent was waived for this retrospective and anonymized study.

### Eligibility criteria

In this single centre study, we screened our musculoskeletal tumour centre’s database from 2000 to 2020 for patients treated for primary bone neoplasms based on ICD codes. The selected tumours were the most frequent in our database: Aneurysmal bone cyst (ABC), chondroblastoma, chondrosarcoma, enchondroma, Ewing sarcoma, fibrous dysplasia, giant cell tumour, non-ossifying fibroma (NOF), osteochondroma, and osteosarcoma. Malignant lesions were verified by histopathology, while benign and intermediate lesions were confirmed by histopathology or discussed in the local tumour board and classified based on radiological features [17]. Clinical and imaging data were retrieved from our HIS and PACS, respectively. The data curation and validation were performed by an orthopaedic resident, (SC) a senior musculoskeletal radiologist (JN), and a data scientist (MG).

### Patients

**Fig. 1:**
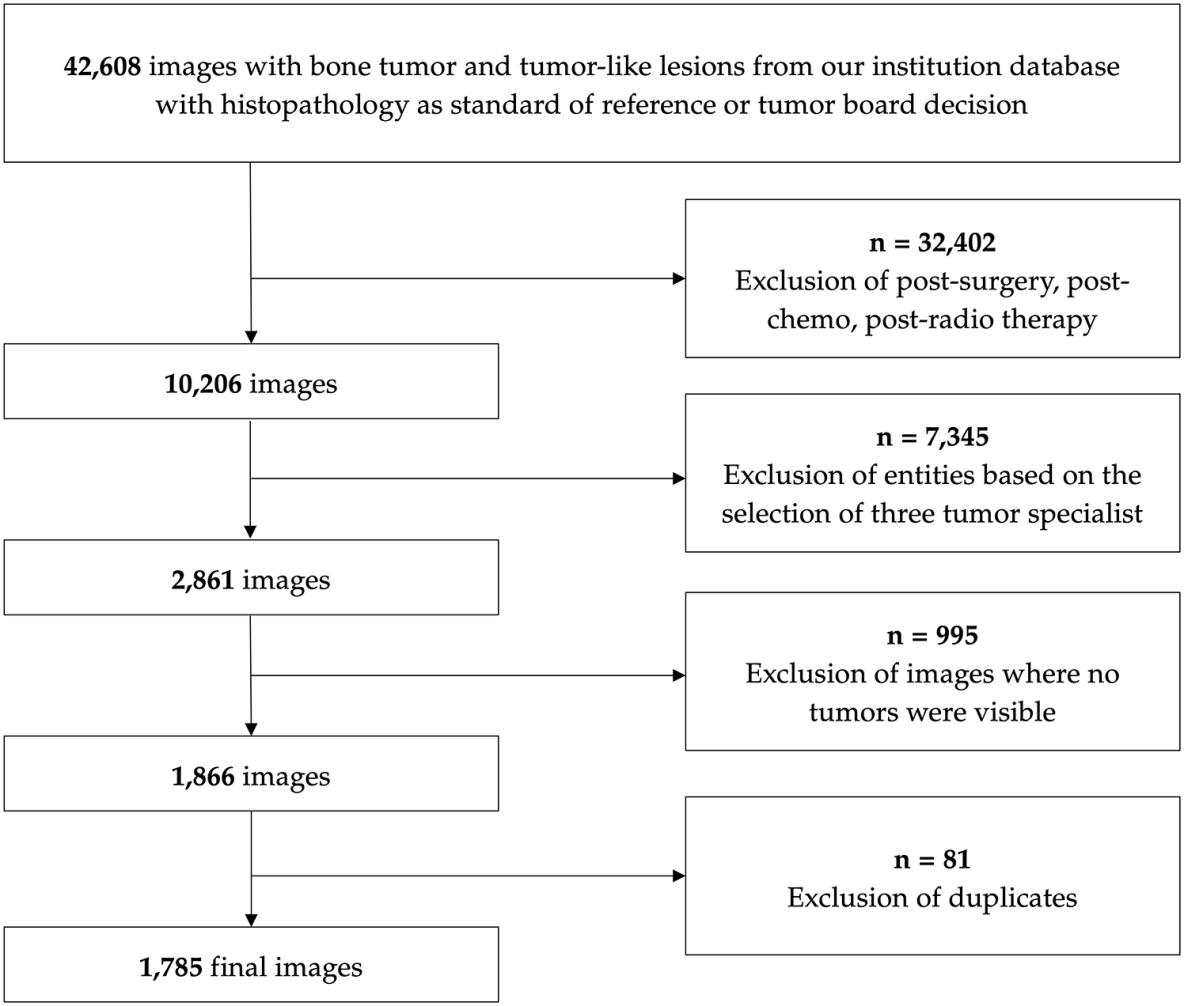
Flow diagram showing the application of eligibility criteria to create a final dataset

We had access to a total of 42,608 radiographic images of bone tumours, tumour-like lesions, and their differential diagnoses, including e.g. osteomyelitis. However, approximately three-fourths of the images were excluded as they were acquired post-surgery, after systemic therapy, or post-radiotherapy. The dataset ultimately included 1,785 images representing ten entities of benign, intermediate, and malignant bone tumours, after excluding 7,345 images that did not meet the criteria (e.g., exostosis, tumour-like lesions). Additionally, 995 images were discarded due to the absence of visible tumours (e.g., wrong angle, artefacts). These images were accompanied by patient metadata (age, affected bone site, tumour position, and gender) from 922 patients. Figure 1 displays the according flow diagram.

### Statistical analysis

Descriptive data follows STROBE guidelines [18], presenting discrete parameters as incidence and percentage ratio, and continuous parameters as mean, standard deviation, median, and interquartile range. The reporting and validation of the prediction model adhere to CLAIM guidelines [19]. Statistical analysis was conducted by two data scientists (MG, > 3 years of experience / FH, > 6 years of experience).

## Image processing

**Fig. 2:**
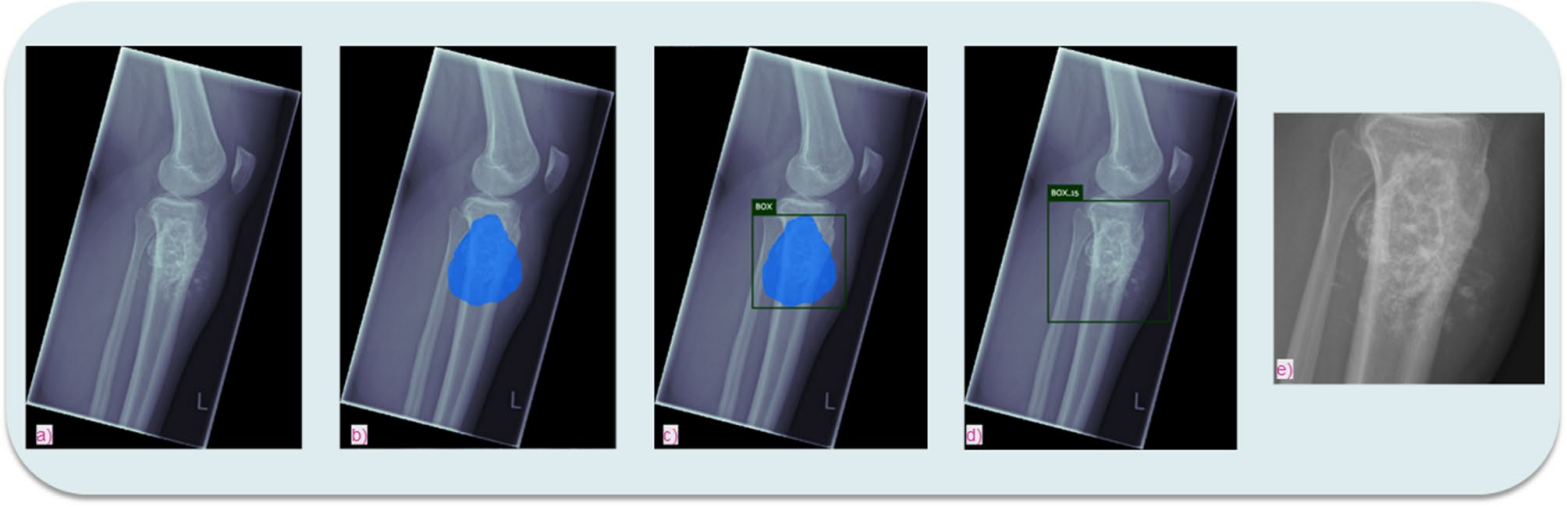
Exemplary creation of bounding boxes focusing the tumorous tissue by the segmentation algorithm of Bloier et al.: **a**) initial image, **b**) segmented tumour, **c**) calculated bounding box, **d**) bounding box with 15% margin to assure all tumour tissue is captured, **e**) cropped image

To accommodate the large size difference between radiographs and the standard DL model input size of 224 × 224 pixels [20, 21], we performed a ROI to remove non-relevant information (Fig. 2). Segmentation masks, created by medical experts (SC, > 6 years of experience / JN, > 8 years of experience), were used for this dataset. An automated segmentation model by Bloier et al. [22] achieved a 99.72% accuracy in predicting these masks. The images were then cropped using a square bounding box around the segmentation mask, with a 15% padding for uncertainty. In the final preprocessing step, the cropped images were converted to standard grey scale and rescaled to 224 × 224 pixels.

### Model development

Our approach combines imaging data and clinical metadata in a single deep learning model. Image features are extracted using a state-of-the-art classification network. Clinical data is processed through fully connected layers with ReLU activation functions and batch normalization, and the resulting features are concatenated. The final softmax layer provides a probability prediction for the entity. During training, the loss is propagated through the entire model, including the image and metadata networks. According to Huang et al. [23], our model configuration is classified as a joint fusion model that is independent of any specific image classification network, chosen because it weighs image and metadata equally.

To compute baseline results, we implemented a XGboost [24] model for classification only with metadata. Further, to compute baseline results for solely imaging data, we compared a ResNet [25] model with the NestT [26] model. ResNet utilizes residual connections to build deeper and more powerful CNNs. NestT, on the other hand, is based on the ViT architecture from the NLP domain, which employs self-attention instead of convolutions [27]. ViT generally outperforms CNNs when datasets are large [28], while NestT performs well even with small datasets [26]. To address the class imbalance in our dataset, we applied data augmentation techniques—such as random rotations, flipping, zooming, and translations, each with a 0.3 probability—to the underrepresented classes, generating additional training samples to improve model generalization. Additionally, we incorporated class weights into the loss function by calculating the inverse frequency of each class. This ensured that misclassifications of rarer tumour classes were penalized more heavily during training, encouraging the model to focus on these underrepresented categories.

The model training and inference were conducted on a DGX Station A100 equipped with four graphical processing units, 64 cores, and 512 GB system memory. The system ran on a Linux/Ubuntu 20.04 distribution. Pre-processing and model implementation were performed using Python 3.9.12, PyTorch Lightning 1.7.1, PyTorch 1.12.1, and the CUDA toolkit 11.3.1. The trained classification model will be made available on GitHub upon publication.

### Outcome and model evaluation

For hyperparameter optimization, we compared the mean validation accuracy using 5-fold cross-validation. The standard deviation was assessed to evaluate model robustness. In our multi-class classification setting, we used macro averaging, which calculates metrics for each class separately and then averages them with equal weight. For evaluating individual class performance, we calculated a confusion matrix. To compare different model architectures and approaches, we assessed the mean test accuracy with 5-fold cross-validation. Additionally, we analysed the ensemble test accuracy, which combines all training data from the five cross-validation folds.

## Model interpretation

Understanding the reasoning behind a model’s prediction is often crucial, but complex models can be difficult to interpret, posing challenges for humans. Deep learning models, in particular, are considered black boxes due to their nested nonlinear structure (29, Samek W. Explainable artificial intelligence: Understanding, visualizing and interpreting deep learning models. arXiv preprint arXiv:1708.08296. 2017.). To address this issue, we implemented SHAP (SHapley Additive exPlanations) introduced by Lundberg et al. [30]. This method calculates the impact of features on individual model predictions. To assess overall performance, we computed average SHAP values across all test samples. As the DeepLiftShap approach does not support Vision Transformers (ViTs), we used the GradientShap algorithm to calculate SHAP values. For instance, the binary-encoded metadata *gender* is represented by two input features, whose sum yields the SHAP value. Similarly, SHAP values for *tumour site* and *position at bone* were computed using the same procedure.

## Results

### Dataset

**Fig. 3:**
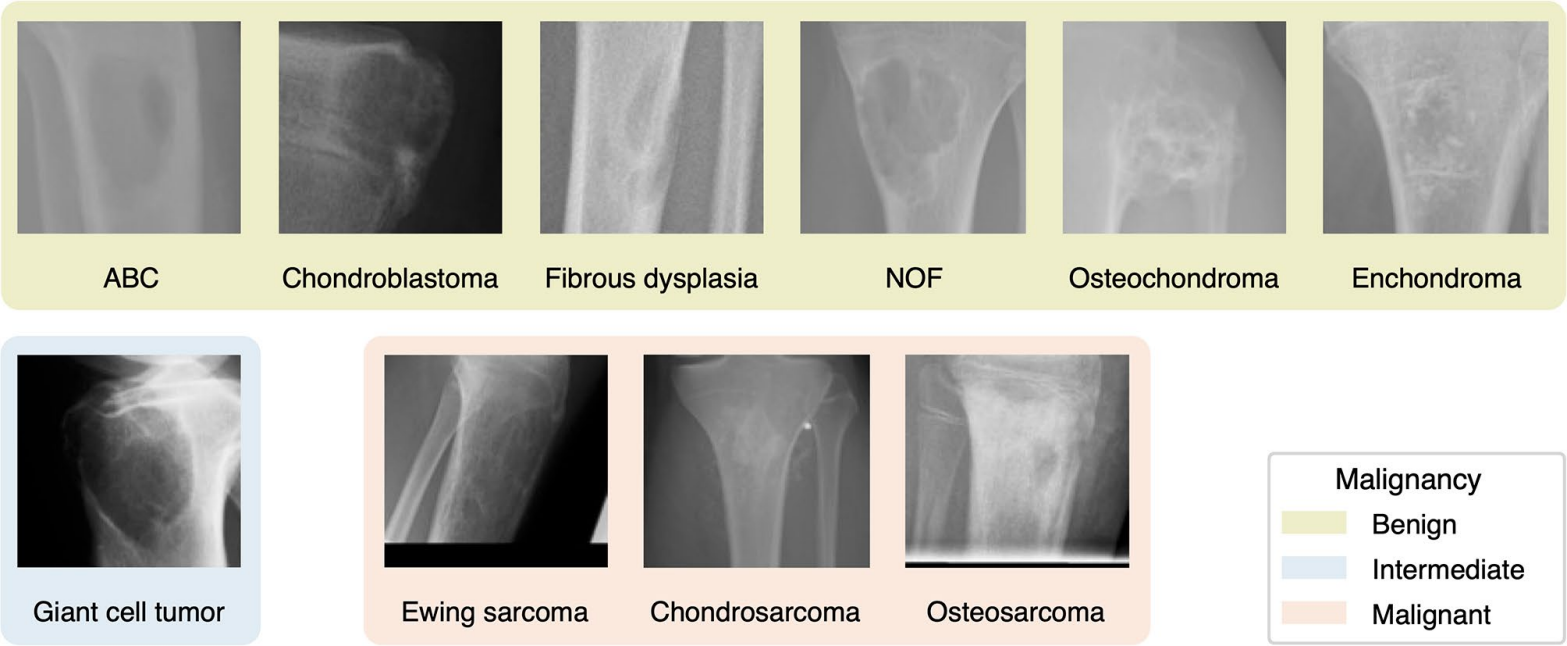
Examples of radiographs of each entity: one example for each entity after pre-processing (cropping, rescaling)

The patients’ mean age was 33.62 ± 18.60. For more information, refer to Table 1. Among the entities in the dataset, osteochondroma was the most common (28.48%), while Ewing sarcoma was the least frequent (0.37%). This indicates an imbalanced dataset with significant variations in sample distribution. The gender distribution was relatively balanced, with females accounting for 45.27% and males for 54.73% of cases, slightly favouring males. The femur was the most common tumour site (36.82%), whereas the os sacrum had only one occurrence (0.12%) in the entire dataset. Additional discrete patient characteristics can be found in Table 2. Figure 3 illustrates one example image per entity after pre-processing.

**Table 1:** Distribution of continuous characteristics (std = standard deviation, IQR = interquartile range)

Age	mean / std	median	IQR
All patients	33.62 ± 18.60	30	30

**Table 2:** Distribution of discrete characteristics with incidence and percentage ratio

Entity (alph.)	#	%
Aneurysmal bone cyst (ABC)	49	6,09
Chondroblastoma	18	2,24
Chondrosarcoma	121	15,1
Enchondroma	180	22,4
Ewing sarcoma	3	0,37
Fibrous dysplasia	37	4,6
Giant cell tumour	50	6,22
Non-ossifying fibroma (NOF)	33	4,1
Osteochondroma	229	28,5
Osteosarcoma	84	10,5
**Gender**	**#**	**%**
Female	364	45,3
Male	440	54,7
**Site** (alph.)	**#**	**%**
Clavicle	7	0,87
Femur	296	36,8
Fibula	42	5,22
Foot	41	5,1
Hand	60	7,46
Humerus	124	15,4
Os ilium	24	2,99
Os ischii	8	1
Os pubis	11	1,37
Os sacrum	1	0,12
Patella	7	0,87
Radius	12	1,49
Scapula	17	2,11
Spine	4	0,5
Tibia	145	18
Ulna	5	0,62
**Total**	**804**	**100**

### Model performance

**Fig. 4:**
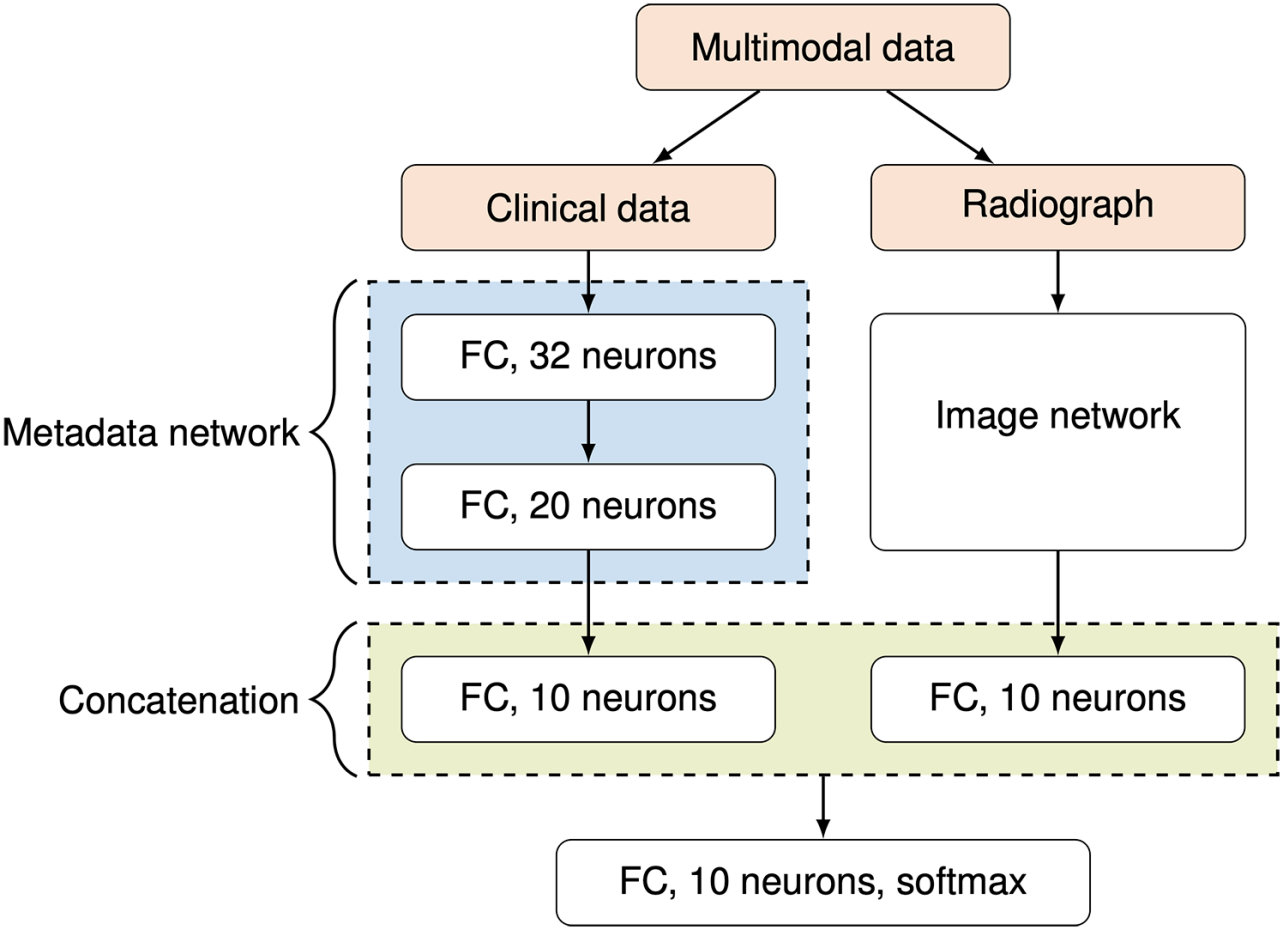
Proposed multimodal model: metadata (clinical data) being processed through a multilayer perceptron, imaging data through common image classification networks and concatenation (fusion) of both in feature space before a final fully connected and softmax layer

Table 3 presents the different tested models, including three “baseline” models (only meta- or only imaging data) and three models based on our proposed architecture. The XGBoost [24] model trained solely with metadata achieved an accuracy of 0.185. For image-only models, we compared ResNet18 [20] and NesT [26], with NesT achieving a higher accuracy of 0.628 ± 0.019 compared to ResNet18’s 0.541 ± 0.029. The NesT architecture with a small model size, a learning rate of α = 0.0005, and a batch size of 16 performed best. Concatenating the features from the NesT model and a separate MLP for metadata, we constructed our multimodal approach. The architecture is depicted in Fig. 4.

.

**Table 3:** Experiment results reporting the test accuracy with standard deviation

Model	Accuracy	F1-Score	Precision	Recall
XGBoost (metadata only)	0.185 ± 0.021	0.180 ± 0.008	0.200 ± 0.019	0.210 ± 0.011
ResNet (images only)	0.541 ± 0.029	0.570 ± 0.026	**0.718 ± 0.029**	0.541 ± 0.029
NestT (images only)	0.628 ± 0.019	0.619 ± 0.020	0.625 ± 0.022	0.628 ± 0.019
ResNet (images and metadata)	0.583 ± 0.018	0.597 ± 0.021	0.686 ± 0.028	0.583 ± 0.018
NestT (images and metadata)	0.643 ± 0.020	0.632 ± 0.025	0.635 ± 0.027	0.643 ± 0.020
NestT ensamble (images and metadata)	**0**,**678**	**0**,**669**	0,668	**0**,**678**

With the multimodal model, we achieved an accuracy of 0.641. When optimizing the hyperparameters, we found that the encoding of the metadata had an impact on performance. For our final model, the features were coded as follows:

- gender: *binary* [male, female].
- age: *ordinal* [1,2,3 etc.]
- site: *one-hot* [clavicle, femur, etc.]
- position: *one-hot* [epiphysis, epi-metaphysis, metaphysis, meta-diaphysis, diaphysis].

All shown results were obtained after hyperparameter tuning.

The average accuracy results from cross-validation were presented. Combining all folds into an ensemble model improved the accuracy by summing up the logits of the five models and applying a softmax layer. The multimodal model showed the highest improvement, achieving a final accuracy of 0.697.

### Comparison of the performance of each entity

**Fig. 5:**
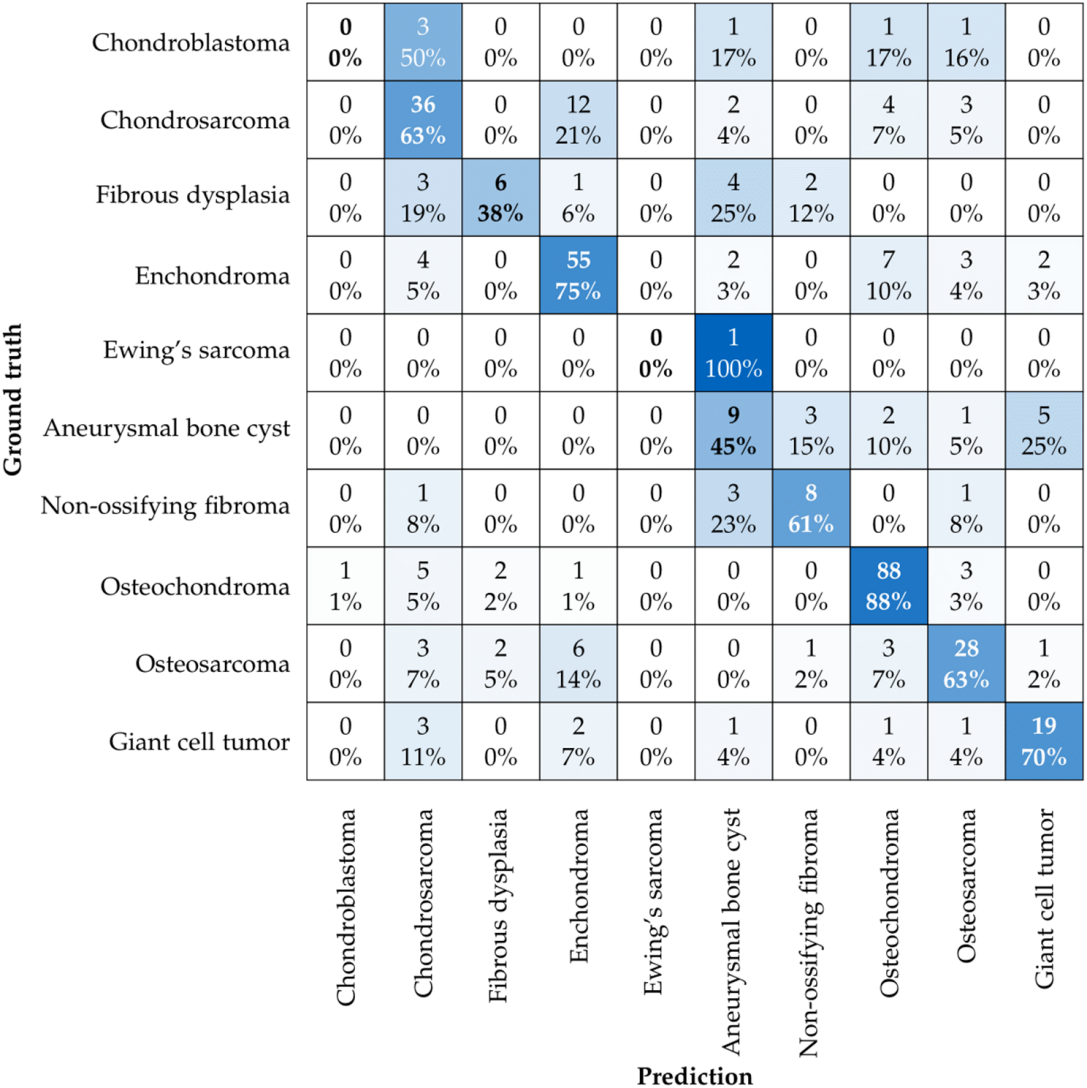
Accuracy scores per entity and respective predictions: The confusion matrix indicates that Chondroblastomas and Ewing sarcomas were classified incorrectly in all instances, resulting in 0% accuracy for these classes. Osteochondromas and enchondromas achieved the best performance with accuracies of 88% and 75%, respectively

Figure 5 presents a confusion matrix that provides the individual accuracy of each bone tumour entity classified by our model. As observed, certain entities such as Chondroblastomas and Ewing sarcomas were not correctly classified in any of the test samples, resulting in 0% accuracy for these classes. This highlights a significant challenge in our model’s ability to generalize for these less frequent tumour types. Conversely, the best performance was noted for osteochondromas with an accuracy of 88% and enchondromas with 75%.

To understand these outcomes better, we performed a bad case study analysis focusing on the instances where the model failed to classify correctly (Table 4). These misclassifications were primarily due to the small number of samples and the high variability within these tumour types, which led to poor generalization. The following table summarizes the misclassified cases for Chondroblastomas and Ewing sarcomas, showing the various false predicted classes.

**Table 4:** Bad case analysis

True class	False predicted class	Number of missclassifications
Chondroblastoma	Enchondroma	4
Chondroblastoma	Giant Cell Tumour	3
Chondroblastoma	Osteochondroma	2
Ewing Sarcoma	Osteosarcoma	2
Ewing Sarcoma	Chondrosarcoma	1
Ewing Sarcoma	Giant Cell Tumour	1

Additionally, we have now included statistical analyses to evaluate the significance of our results. P-values were calculated to compare the performance of our multimodal model against the baseline models using a paired t-test. The results demonstrate that our proposed model significantly outperforms the baseline models with p-values < 0.05, thus confirming the statistical significance of the improvements observed (Table 5).

**Table 5:** P-values for model comparison

Model	*p*-value
XGBoost (metadata only) vs. Multimodal	< 0.001
ResNet (images only) vs. Multimodal	0.003
NesT (images only) vs. Multimodal	0.010
ResNet (images + metadata) vs. Multimodal	0.005
NesT (images + metadata) vs. Multimodal	0.021

### Explainability through SHAP

**Fig. 6:**
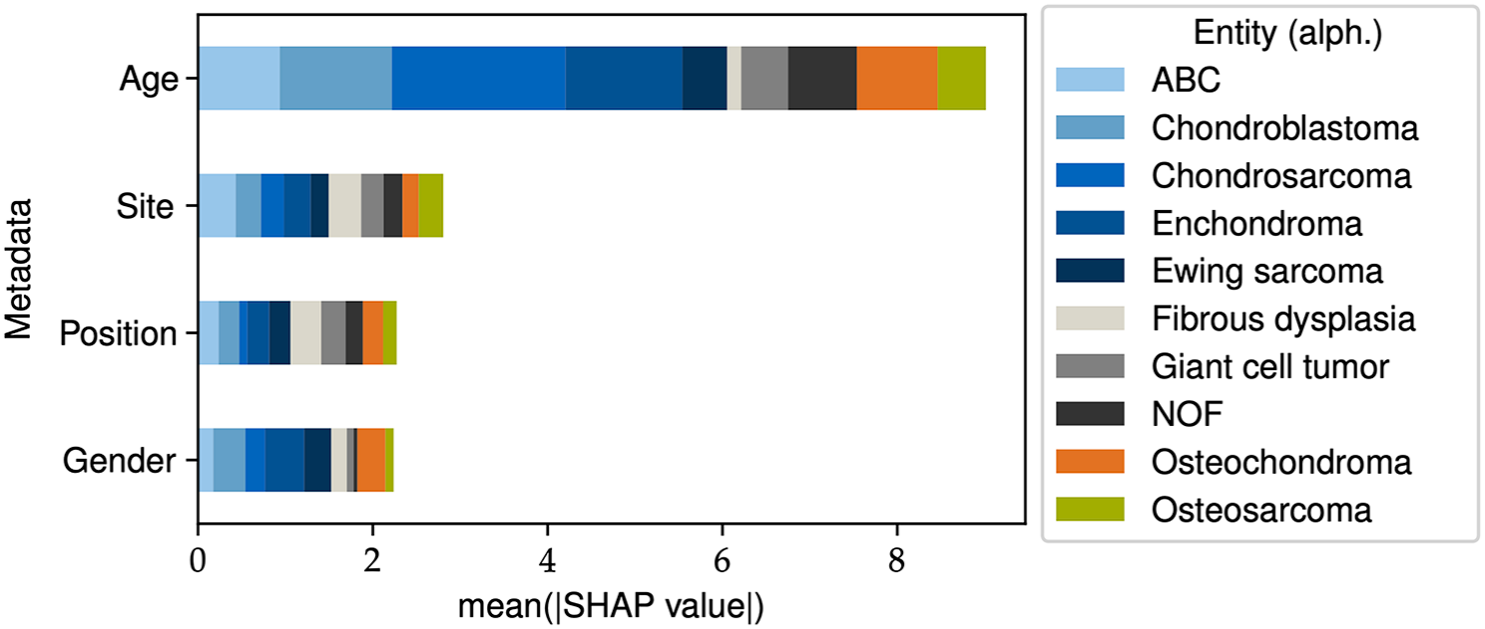
SHAP results for the best-performing NesT model: the contribution of the features based on the entity measured in SHAP values. The values are calculated based on the test dataset, taking the mean of the absolute values (age = patient’s age, site = affected bone, position = position of the tumour at the bone, gender = gender of the patient)

**Fig. 7:**
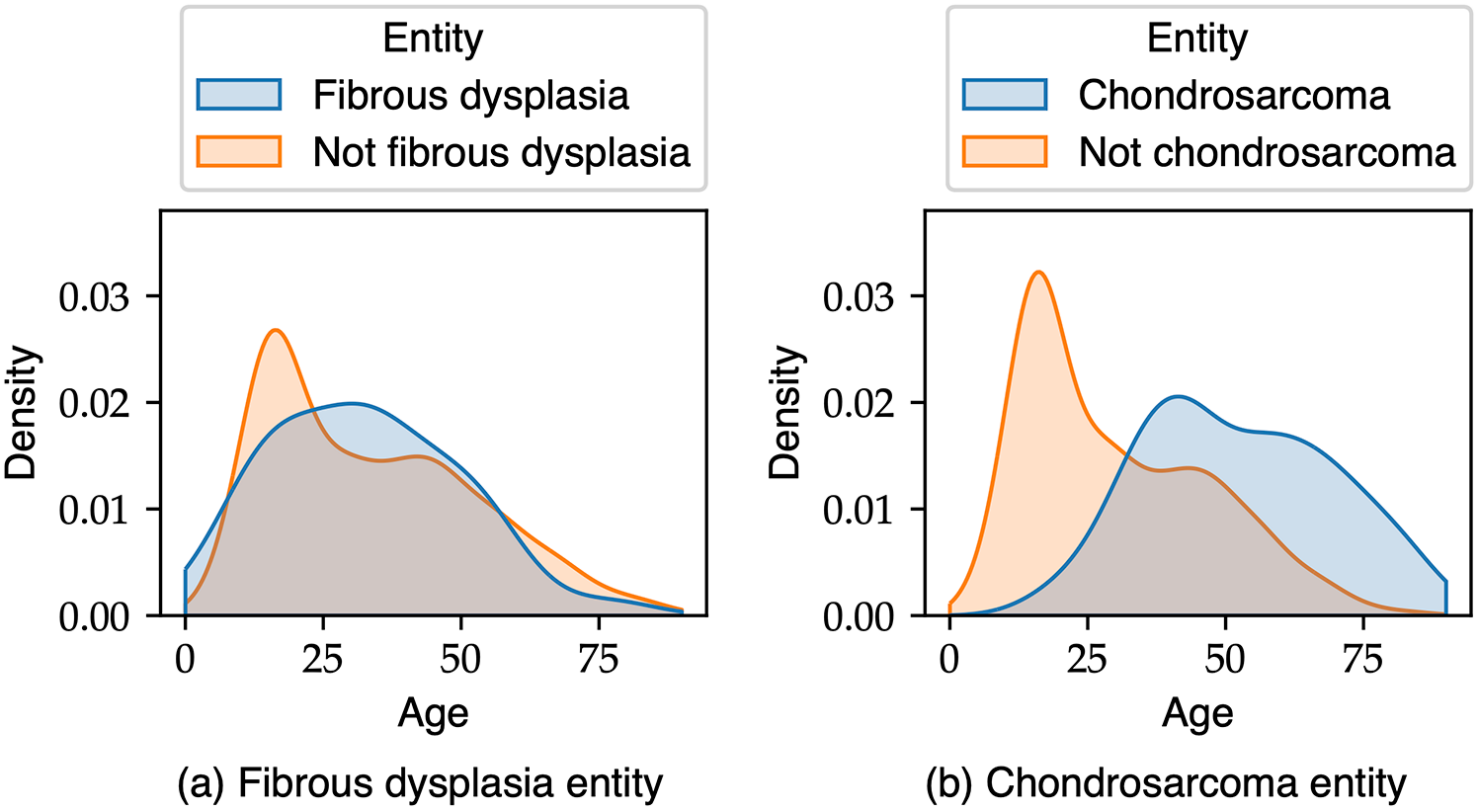
Age distribution for two entities compared to the overall distribution of the other entities. Normalization is calculated separately for both groups (entity/not-entity), since the not-entity group contains many more samples

We used the SHAP framework to evaluate the impact of metadata on the best-performing NesT model, as introduced by Lundberg et al. [30]. In Fig. 6, the averaged SHAP results are visualized. Age was found to have the highest impact on the prediction, while the contribution of other metadata features was similar. Additionally, the influence of metadata on output predictions varied for each entity. Chondrosarcoma showed the highest SHAP value for age, while fibrous dysplasia had the lowest. To gain further insights, we examined the age distributions for these entities, comparing them with the rest of the dataset. Figure 7a illustrates the age distribution for fibrous dysplasia, and a similar comparison is made for chondrosarcoma.

## Discussion

The main finding of this study was the successful development of a transformer and MLP-based model for classifying ten primary bone tumour entities. The inclusion of clinical metadata, in addition to radiography, showed improvements in classification accuracy. While the results are not yet ready for clinical application, they highlight the potential of addressing the complex diagnostic classification task by increasing data quality and quantity. Integration of more comprehensive data, as suggested in literature [1, 3, 7], could further enhance the model’s performance. Additionally, the implementation of SHAP helped provide insights into the impact of metadata and interpret the black-box nature of DL models. It is important to note that the age plot for fibrous dysplasia and other entities showed a significant intersection, making it challenging to extract information based on age alone for this entity. Conversely, the age plot for chondrosarcoma displayed a larger impact according to SHAP values, with less intersection with other entities (Fig. 7b). This suggests that age is a useful feature for distinguishing the presence of chondrosarcoma.

The bad case study analysis reveals several factors contributing to the misclassifications. For Chondroblastomas and Ewing sarcomas, the limited number of samples and the high variability within these tumour types were significant challenges. Additionally, visual similarities in radiographs and overlapping radiographic features contributed to these misclassifications. For example, Chondroblastomas were often misclassified as Enchondromas and Giant Cell Tumours due to their visual similarity and insufficient distinct features captured in the metadata. Ewing Sarcoma was sometimes confused with Osteosarcoma and Chondrosarcoma due to their similar radiographic appearance and high intra-class variability.”

A major limitation of this study is the size of the dataset. While having 1,785 radiographs is significant for rare primary bone tumour entities, the average of 178 samples per class is relatively low considering the heterogeneity of bone lesions and the requirements of deep learning. This limited dataset results in class imbalance, which is common in medical datasets. The most common entity (osteochondroma) has 501 occurrences, while the least common entity (Ewing sarcoma) has only 6 occurrences. The underrepresentation of less frequent classes can lead to overfitting and poor generalization [28]. Another limitation is the lack of evaluation using external data. Although around 10% of the dataset consists of radiographs from external sources, such as general practitioners and external radiologists, it is necessary to test the model on additional external data to assess its generalizability [31]. This assessment is crucial before considering the model’s suitability for clinical use [32].

Several prior studies have explored the classification of bone tumours using imaging data [33, 34] or have demonstrated multimodal approaches for integrating imaging and tabular data in medical classification [28, 35]. For instance, von Schacky et al. [34] developed a multitask deep learning (DL) model capable of simultaneously detecting, segmenting, and classifying bone lesions, comparing its performance against radiologists of varying experience levels. The overall task of classifying bone lesions and the specific entities examined in their study were similar to those in our research. While their model achieved a classification accuracy of 43.2%, a musculoskeletal radiologist achieved 58.6% accuracy in classifying bone lesions on an entity level. Although our metric values were significantly higher, von Schacky et al. had to contend with a lower sample ratio per class, fewer patients, and thus a smaller overall dataset. Their study primarily focused on a multitasking model and comparison with human experts, while our emphasis cantered on integrating clinical metadata in conjunction with imaging data using state-of-the-art techniques. Nonetheless, their study underscores the intricate nature of accurately identifying bone neoplasms for both DL models and clinical professionals. In a similar study, Liu et al. [36] proposed a deep learning-machine learning model for classifying bone tumours using patient clinical metadata and radiographs. They collected 982 radiographs from 643 patients, incorporating clinical metadata such as age, gender, and location. Their approach involved using an Inception V3 model to process imaging data and fusing its output with clinical features to train an XGBoost model. Their fusion model achieved a top macro area under the curve of 0.872, outperforming five radiologists by 0.819. The main difference between their study and ours is their focus on predicting tumour malignancy, while we aimed to classify ten tumour entities. The classification task differs due to the number of classes and sample sizes. Our fusion approach captures both image and metadata information, while Liu et al. combined DL model probabilities with metadata before using a secondary model. We hypothesize that our approach better aligns with the clinical algorithm used by radiologists and surgeons, as it simultaneously evaluates metadata and imaging data for comprehensive and accurate bone tumour assessment, leading to improved performance. Xu et al. [37] presented a notable study that employed multimodal data and a fusion approach for accurate differential diagnosis of skin tumours. They introduced a transformer model capable of leveraging multimodality imaging and non-imaging data to enhance diagnostic performance. Their approach involved integrating a cross-modality fusion module with a transformer-based multimodal classification system, enabling the fusion of data from multiple sources. The dataset used in their study encompassed dermoscopy, clinical imaging, and patient metadata. To evaluate the effectiveness of their proposed model, Xu et al. conducted experiments on both a public dataset (Derm7pt, 1,011 cases) and an in-house dataset (5,601 cases). The results were highly promising, surpassing the state-of-the-art performance with a 2.8% increase and achieving an impressive accuracy of 88.5%, respectively. In comparison to our model, the approach described by Xu et al. demonstrated the capability to incorporate multimodal imaging in addition to metadata. While “remixing” metadata within disease classes yielded positive results in their specific domain, we posit that in our case, metadata and image features are closely intertwined and should not be interchangeably treated. Nevertheless, it is important to note that no existing model, to the best of our knowledge, has proposed a multimodal approach that integrates both imaging and patient-specific metadata for bone tumour classification.

The framework with a transformer model and MLP, combined through feature join for image and metadata processing, is transferable to other scenarios where integration of different types of information is crucial for decision-making. The retrospective dataset, spanning 20 years and including diverse patient populations and imaging devices, ensures a lack of strong bias and good generalizability. However, although the dataset is considerable for rare bone tumours, it is not extremely large in terms of DL. To ensure broader generalizability, a larger dataset should be collected in the future.

The proposed model’s results do not yet have direct clinical relevance, but the increased accuracy achieved through state-of-the-art methodology shows promise. Enriching the imaging dataset with clinical metadata brings AI models closer to the approach of human experts. These promising results, along with other applications of AI models in medicine, could raise awareness among domain experts. Optimal AI model performance relies on domain experts supporting the collection of complete, accurate, and comprehensive medical data, as data quality and quantity are vital factors.

## Conclusion

We developed a novel fusion model combining NesT and MLP to integrate imaging data and clinical metadata for bone tumour classification. By enriching the imaging dataset with patient-specific clinical metadata, such as age, gender, tumour position, and site, we improved performance and surpassed similar studies. This approach aligns with current clinical diagnostic workflows, where imaging data and patient characteristics are evaluated together for tumour assessment. While the results are not yet suitable for clinical application, we believe that structured data collection can further enhance our model’s performance, making it a valuable tool for radiologists, surgeons, and general practitioners in bone tumour assessment.

## Data Availability

The data that support the findings of this study are not openly available due to reasons of sensitivity and are available from the corresponding author upon reasonable request. Data are located in controlled access data storage at Klinikum rechts der Isar, Technical University of Munich.

## Abbreviations

AI: Artificial intelligence
DL: Deep learning
NesT: Nested Hierarchical Transformer
MLP: Multilayer perceptron
SHAP: Shapley additive explanations
ViT: Vision transformer
NLP: Natural language processing
